# The expressions of AMACR and iNOS in prostate adenocarcinomas

**Published:** 2013-04

**Authors:** Tumay Ozgur, Esin Atik, Sibel Hakverdi, Mehmet Yaldiz

**Affiliations:** 1 Tumay Ozgur, MD, Assistant Professor, Mustafa Kemal University, School of Medicine, Department of Pathology, Hatay, Turkey; 2 Esin Atik, MD, Associate Professor, Mustafa Kemal University, School of Medicine, Department of Pathology, Hatay, Turkey; 3 Sibel Hakverdi, MD, Assistant Professor, Mustafa Kemal University, School of Medicine, Department of Pathology, Hatay, Turkey; 4 Mehmet Yaldiz, MD, Professor, Mustafa Kemal University, School of Medicine, Department of Pathology, Hatay, Turkey

**Keywords:** Prostate, adenocarcinoma, AMACR, iNOS

## Abstract

***Background and Objective:*** Prostate cancer is the second leading cause of death in men. The localized disease often responds to conventional therapies like androgen ablation via castration and/or administration of chemical inhibitors but advanced disease resistant to any curative therapies is still challenge for investigators. There are increasing efforts to enhance the possibility of finding positive and sensitive immune markers for diagnosing and treating prostate cancer. The aim of this study was to investigate the expression patterns of AMACR and iNOS in prostate adenocarcinomas with different histopathologic grade.

***Methodology:*** We applied immunohistochemical markers; AMACR and iNOS. Formalin-fixed parafin embedded tissues of 64 prostate needle biopsy specimens diagnosed as prostate adenocarcinoma between 2005-2010 years were included in the study.

***Results:*** AMACR expression were found in 58 (90.6%) and iNOS expression in 54 (84.4%) of 64 prostate adenocarcinomas. No significant relationship of AMACR and iNOS was obtained (p>0.05). There was no significant correlation of histopathologic grade of the tumors with AMACR and iNOS expression (p>0.05).

***Conclusions:*** The expression of AMACR and iNOS might be important diagnostic immune markers for prostate adenocarcinomas especially in needle biopsies where the quantity and quality of tissue are limited.

## INTRODUCTION

Prostate adenocarcinoma (PA) is a frequent non-cutaneous neoplasm in Western countries.^1^ The localized disease often responds to conventional therapies like androgen ablation via castration and/or administration of chemical inhibitors but advanced disease resistant to any curative therapies is still challenge for investigators.^2^There are increasing efforts to increase the possibility of finding positive and sensitive immune markers for diagnosing and treating PA.^2^^,^^3^ Alfa-methyl-acyl-CoA racemase (AMACR) is an enzyme that functions in peroxisomal beta oxidation of dietary branched chain fatty acids and C27 bile acid intermediates.^4^^-^^6^

Increased fatty acid synthesis and the use of branched fatty acids may play important roles in development and progression of PA.^7^ AMACR is overexpressed in premalignant and malignant lesions of the prostate compared within the normal prostate.^5^^,^^8^^-^^10^ The levels of AMACR elevation continues as PA initiates to higher grades and stages.^7^ There are studies that shows that the chromosomal region for AMACR (5p13) is the candidate region of gene and AMACR gene polymorphism is frequent in PAs of families that have hereditary PA.^6^^,^^11^

AMACR is essential for optimal growth of prostate carcinoma cells in vitro and this enzyme has the potential to be a complementary target with androgen ablation in prostate carcinoma treatment.^7^

Nitric oxide (NO) is synthesized from L-arginine by isoenzymes called NO synthases (NOS) in a variety of tissues. NOS are composed of subtypes according to naming, constitutive and inducing roles; neuronal NOS (nNOS)and endothelial NOS (eNOS) are responsible for neurotransmission and vasodilatation while inducible NOS (iNOS) conducts macrophages and tumor-induced immunsupression.^12^^,^^13^ NOS is expressed by several tumors like human ovarian, stomach and breast carcinoma.^14^ There are reports defining selective expression of iNOS in human prostate carcinoma in the literature.^15^^,^^16^

Evidences show that iNOS activity has positive correlation with tumour progression by stimulation of angiogenesis and increased mutagenesis which makes this molecule a therapeutic target for the treatment of cancer.^2^^,^^3^^,^^17^

To the best of our knowledge, there is no previous investigations on the combined analyse of these two markers in needle biopsies diagnosed as PA. The goal of our study was to investigate AMACR and iNOS expressions in PAs diagnosed in needle biopsies at our institute and correlate them with tumor grade.

## METHODOLOGY

This was a retrospective archive study including 64 male patients who were diagnosed as PA in the Pathology Department of Hatay Mustafa Kemal University, Turkey between 2004-2010. Only patients that were diagnosed with needle biopsies were included in this study. The ethical committee on human research at our institution approved the protocol for all human research. The haematoxylin-eosin (HE) stained cross sections of the cases have been re-evaluated for histopathology on the basis of the grading PAs. The prepate representing the tumor in the best manner was selected.

Section of 3-4 mm thickness was cut from the paraffin blocks of these preparations and then were de-paraffinized and rehydrated through a graded series of alcohol, microwave antigen retrieval method was used, followed by incubation with AMACR (Prediluated Polyclonal Rabbit Anti-Human P504S protein, Biocare Medical, Concord, CA, USA.) and iNOS (Prediluated polyclonal Rabbit Anti-Human INOS protein, Genetex, San Antonio, Texas, USA.) Immunohistologic staining (IHS) was applied. Atrophic glands for AMACR and endothelium adjacent to prostatic carcinoma tissue for iNOS in the same section have been accepted as internal positive controls.

Immunoreactivity was scored by a semiquantitative scoring method. The expressions of iNOS and AMACR were evaluated in the entire section at a magnification of X 400 by an Olympus BX53 light microscope. Membranous staining patern for AMACR, cytoplasmic staining patern for iNOS have been accepted while evaluating the tumors.

The staining intensity of all cases were classified in 4 categories according to Rubin et al. and Baltaci et al.^9^^,^^16^ Group 0 no visible staining, Group 1 weak positive, Group 2 moderate positive, Group 3 strong positive staining.

***Statistical Analysis:*** Statistical evaluations were performed using the “SPSS 13,0 for Windows” packet program and p<0.05 was considered statistically significant. For the comparison of the findings, Pearson Chi-Square and Spearman correlation tests were performed.

## RESULTS

Of the 64 cases enrolled in the study, 44 were Grade 2 (68.8%), 17 Grade 3 (29%) and 2 Grade 1 (3.1%) according to the PA scoring system by Gleason.

**Fig.1:**
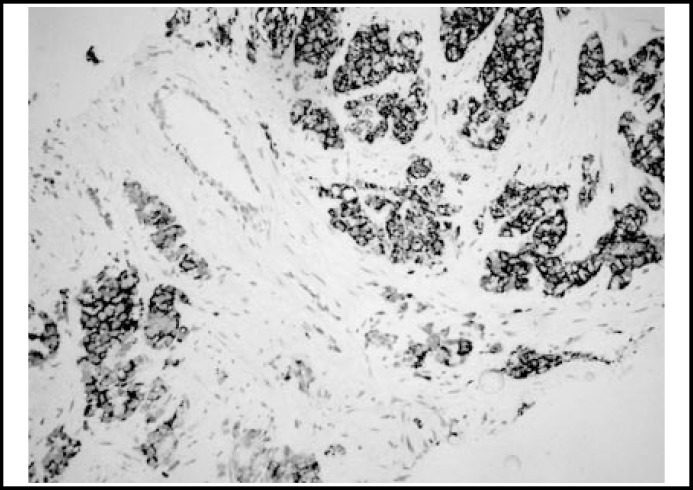
Strong and membranous staining (AMACRX200

**Fig.2:**
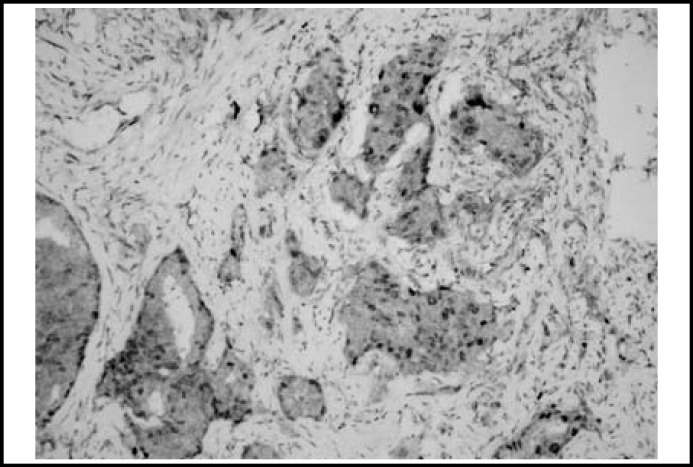
Moderate and cytoplasmic staining (iNOSx200

Descriptive statistics of histopathological diagnose, AMACR and iNOS are shown in Table-I. Almost all the prostate carcinomas were positive for AMACR and iNOS in varying intensity (Fig. 1 and 2).

**Table-I:** Distribution of positive immunostaining for AMACR and iNOS among all the Groups and Grades in prostate needle biopsies

*Immunstains*	*Groups (n/%)*				*Grade (n/%)*		
	Group 0	Group 1	Group 2	Group 3	Grade1	Grade 2	Grade 3
AMACR*	6/9.4	5/7.8	24/37.5	29/45.3	2/3.4	41/70.6	15/25.9
iNOS	10/15.6	33/51.6	19/29.7	2/3.1	2/3.7	38/70.3	14/25.9

AMACR vs iNOS; *p>0.05

AMACR expression has been found in 58 (90.6%) and iNOS expression in 54 (84.4%) of 64 PAs. AMACR staining distributed as Group 3 in 45.3% (29), Group 2 in 37.5% (24), Group 1 in 7.8% (5) and Group 0 in 9.4% (6) of PA cases. iNOS staining distributed as Group 3 in 3.1% (2), Group 2 in 29.7% (19), Group 1 in 51.6% (33)and Group 0 in 15.6% (10)of PA cases. No significant relationship of AMACR and iNOS expressions have been obtained (p>0.05).

There was no significant correlation of histopathologic grade of the tumors with AMACR and iNOS expressions (p>0.05).

## DISCUSSION

There are increasing efforts to enhance the possibility of finding positive and sensitive immune markers for detecting prostate cancer since PSA screening has limited specifity. These markers would be useful especially in diagnosing carcinomas that are in small focis in needle biopsies.^18^ Tissue marker identification of the latent and incidental cancers is important to identify differences between significant or aggressive and insignificant or inactive cancers.^19^

We need not only more specific prostate cancer markers but also better markers of biologically relevant disease. AMACR overexpression is an early event in prostate carcinogenesis and indicate malignant transformation.^7^^,^^20^ AMACR could be one of the immune markers that have role in distinguishing ordinary and aggressive prostate cancer and this would improve our understanding of prognostic values.^5^

There are several studies that investigated AMACR expression patterns in the literature.^19^^,^^21^^-^^23^ AMACR is a valuable diagnostic marker because of its persistent and strong expression in case of needle biopsies when the tissue is limited.^18^ In a study conducted by Sreekumar et al. they have found that PSA was non-specific and all men showed immun response to PSA free from cancer. But AMACR had more sensitivity and specifity for cancer patients compared to control group.^24^

In our study we have found strong and diffuse positive AMACR expression in the majority of our cases. Similar to our findings, Santinell et al. evaluated AMACR, Ki-67 and topoisomerase alpha II in PA and determined increased proportions of positive cells from atrophy through high grade PIN and PA.^19^

We have determined no significant relationship with tumor grade and AMACR expression patterns (p>0.5). Similar to our findings, Rubin et al investigated AMACR expression in 128 prostate adenocarcinomas from different histopathologic grade and found no association with AMACR staining intensity and Gleason score.^25^

Beside this they have identified that high grade PIN also demonstrated AMACR protein expression and noted that AMACR alone would not be a useful marker alone especially in diagnostically challenging cases. Therefore, combined panels of immunstains could be more appropiate in differentiating these lesions.^25^

There are several studies which have assessed INOS activity by immunohistochemistry and RNA analysis invivo and invitro but questions about the generation if NO by INOS with its role in tumorogenesis and tumor prognosis still remain.^26^^-^^28^ iNOS might be a challenging protein for the PA patients needing pre and post-operative indicators for the management of their diseases.^3^^,^^28^^,^^29^

We determined positive staining of iNOS in various paterns in our series but there has been no statistical significant relationship between tumour grade and staining degree. There are studies in line with our findings in the literature. Klotz et al studied iNOS expression in PAs and benign hyperplasia and detected positive immunostaining in all PAs sections but they could not find any difference between the density of immunostaining and tumor grade.^15^ In addition to this, Baltaci et al detected homogenous staining of INOS in PAs. But there has been no correlation between the Gleason score and the degree of iNOS immunreactivity.^16^

Similar to these studies, Aaltomaa et al searched for iNOS expression in 82 patients with local prostate cancer and there was weak or strong expression of iNOS in 25 (31%) and 56 (68%) of the patients but they also could not find any significant association with tumor grade and iNOS expression patern.^26^ These could be explained by the small number of groups and narrow range of Gleason scores.

The main limitations of our study are represented by the small number of cases studied and lack of a control group of prostate glands without cancer for comparison of marker expression. However, our data might have important clinical significance and a message; the combination of multipl markers may improve the sensitivity of the diagnosis and these markers are important for follow-up and obtain biologic nature of these lesions. Further studies with larger series with immunohistochemistry and molecular biology are needed to determine the role of iNOS and AMACR in the pathogenesis of PA.
